# Effective testing of personal protective equipment in blast loading conditions in shock tube: Comparison of three different testing locations

**DOI:** 10.1371/journal.pone.0198968

**Published:** 2018-06-12

**Authors:** Maciej Skotak, Eren Alay, James Q. Zheng, Virginia Halls, Namas Chandra

**Affiliations:** 1 Department of Biomedical Engineering, New Jersey Institute of Technology, Newark, NJ, United States of America; 2 Program Executive Office—Soldier, United States Army, Fort Belvoir, VA, United States of America; University of Illinois at Urbana-Champaign, UNITED STATES

## Abstract

We exposed a headform instrumented with 10 pressure sensors mounted flush with the surface to a shock wave with three nominal intensities: 70, 140 and 210 kPa. The headform was mounted on a Hybrid III neck, in a rigid configuration to eliminate motion and associated pressure variations. We evaluated the effect of the test location by placing the headform inside, at the end and outside of the shock tube. The shock wave intensity gradually decreases the further it travels in the shock tube and the end effect degrades shock wave characteristics, which makes comparison of the results obtained at three locations a difficult task. To resolve these issues, we developed a simple strategy of data reduction: the respective pressure parameters recorded by headform sensors were divided by their equivalents associated with the incident shock wave. As a result, we obtained a comprehensive set of non-dimensional parameters. These non-dimensional parameters (or amplification factors) allow for direct comparison of pressure waveform characteristic parameters generated by a range of incident shock waves differing in intensity and for the headform located in different locations. Using this approach, we found a correlation function which allows prediction of the peak pressure on the headform that depends only on the peak pressure of the incident shock wave (for specific sensor location on the headform), and itis independent on the headform location. We also found a similar relationship for the rise time. However, for the duration and impulse, comparable correlation functions do not exist. These findings using a headform with simplified geometry are baseline values and address a need for the development of standardized parameters for the evaluation of personal protective equipment (PPE) under shock wave loading.

## Introduction

The survival rate among injured warfighters in recent Operation Enduring Freedom and Operation Iraqi Freedom exceeds 90% [1–3]. It is a dramatic improvement among casualty survival compared to previous military conflicts, and it is attributed to advancements in the following areas: 1) battlefield hemorrhage control, 2) far forward trauma surgical and resuscitation techniques, 3) rapid evacuation to higher medical capability facilities and 4) advances in body armor [1, 3]. Among these factors, the impact of the helmet design is not well-established and received relatively little importance as a factor directly implicated in blast mitigation, in spite high prevalence of mild Traumatic Brain Injury among military personnel in the 2000–2017 period (82.3% as of Aug. 2017) [4]. Traditionally, the perception of the helmet is that it is personal protective equipment (PPE) against the ballistic impact of high-energy projectiles and shrapnel, and thus the blast protection was not considered as top priority until recently [5–8].

The evolution of military ballistic helmets to meet ever increasing demands of the battlefield lead to the development of advanced composite materials which outperform their steel-based counterparts used in the WWI to Vietnam War era [5]. The advent of these contemporary modern designs was possible with the invention of high-performance fiber-reinforced polymer-matrix composites, which allow weight reduction without adverse impact or even improvement of the ballistic performance [5, 9]. Typical mechanical properties of these high-performance polymers are the following: 1) failure strength (2.7–5.6 GPa), 2) axial modulus (60–340 GPa), 3) failure strain (0.011–0.045), and 4) relatively low densities (970–1710 kg/m^3^) [10]. The mass-based specific energy absorption capacity is important selection criterion, which favors high molecular weight polyethylene (50–65 kJ/kg) based designs over aramid (25–35 kJ/kg) composites [10, 11]. The ballistic performance of armor materials can be further augmented by preparation of highly ordered structures made of high-performance fibers. Work in this area is relying heavily on the use of numerical [10, 12, 13] and phenomenological models [11, 14].

Evaluation of military helmets under blast loading conditions is still relatively unexplored and developing field. There are no existing field data on the effectiveness of helmets used in the theatre just because of the implementation of the pressure sensors to measure the pressure inside of the helmet is both impractical and technically challenging. Only until recently the surface pressure measurements in active military personnel via deployment of the Blast Gauge™ was realized [15]. Hence, the evaluation of the performance of helmets rests exclusively in the domain of laboratory research. In this context, numerical simulations have dominated the research area thanks to the availability of high-resolution CT and MRI scans, which allow reconstruction of high fidelity 3D computer models with segmentation of specific anatomical details of the human head [16–18].

Typically two related areas of interest are investigated using numerical models: 1) the effects of stress waves propagation in the brain originating from blast wave transmission through the unprotected head [18–20], and 2) the protective properties of helmets [6–8, 21–26] or other PPE designed to mitigate blast effects in the craniofacial area [23]. Numerical simulations are supplemented by experimental data, most often either surface pressure measured on the helmet/head [6, 7, 20], or intracranial pressure [19, 26], to validate the model. Experimental work, which used surrogate anthropometric models, also requires validation via application of surface and intracranial pressure measurements in post-mortem human specimens (PMHSs) [24, 27, 28]. These studies are relatively rare, difficult to execute and impractical for the evaluation of PPE under blast loading conditions. The alternative is to use human head surrogates, which replicate the geometry but are made of non-biological materials [7, 19, 20, 29, 30].

The methodologies for helmet testing in the blunt and ballistic impact are well established, but there are no such tests methodologies available for helmets under blast loading. Shock tubes offer the best mode of testing new and existing helmet designs as means of effective blast protection. However, there are no existing standards for blast testing of helmets, and this work aims to examine the effect of test location on the efficacy of helmet testing. We performed experimental surface pressure mapping on the headform exposed to three blast overpressures at three different locations: inside, end and outside. The goal is to evaluate whether there exists the correlation between four characteristics (peak overpressure, rise time, duration and impulse) of the pressure pulse measured at 10 discrete locations on the headform, and at three test locations. Ultimately, we are seeking the answer to the question whether the comparison between different test setups is possible, or alternative solutions for PPE blast evaluation are necessary. This work is an initial step towards standardization of the PPE testing.

## Materials and methods

### The shock tube

**Fig 1:**
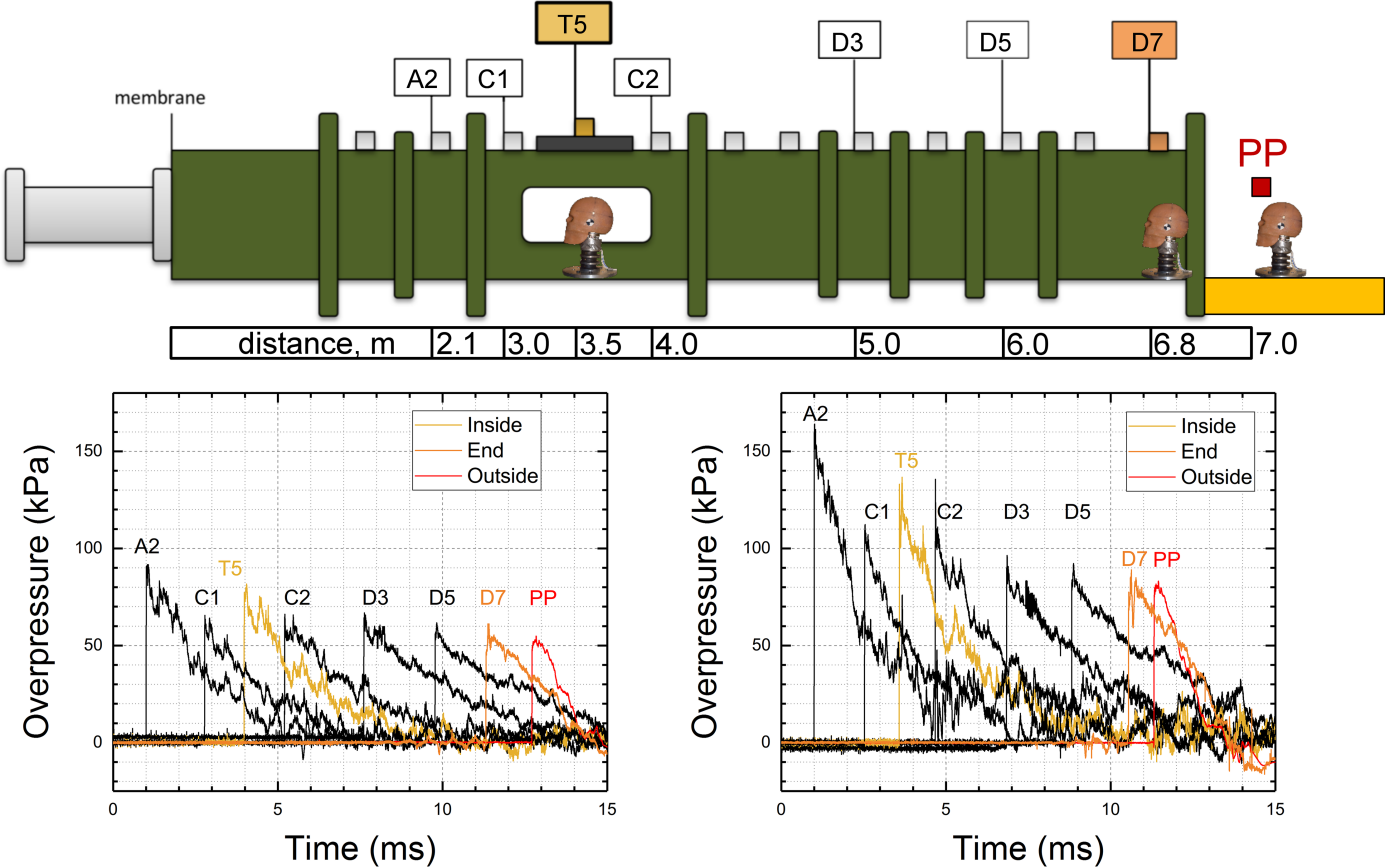
Schematic representation of the 28-inch shock tube illustrating sensor notation and their distribution. The distance between sensors and the membrane mounting port is indicated. The headform was mounted in three different locations: 1) T5 (denoted as “Inside”), 2) D7 (denoted as “End”) and 3) PP (denoted as “Outside”). Bottom panel presents the evolution of the shock wave profile as a function of sensor location for two nominal BOPs of approximately 70 kPa (T5, left) and 140 kPa (T5, right). The arrival times were adjusted for the clarity of presentation. Profiles for T5, D7 and PP locations are highlighted in color.

The square cross section (0.71 x 0.71 m) shock tube was used in all experiments. This device is located at the Shock Wave Testing facility at the Center for Injury Biomechanics, Materials and Medicine (CIBM^3^) at the New Jersey Institute of Technology campus. There are four principal components of this shock tube (Fig 1): 1) adjustable volume breech, 2) transition section, 3) the test section including bullet-proof glass observation station for high-speed video monitoring, and 4) the catch tank designed to contain exhaust gases and dissipate the noise (not shown). The catch tank was not used in the exposures where headform was mounted in the outside test location. We used compressed helium (ultra-high purity, 99.99%, Airgas, Oakland, NJ) as driver gas for all experiments. Helium was allowed to flow into the breech separated from the main body of the shock tube with a stack of Mylar membranes (Grafix, Cleveland, OH) until mechanical failure. We used circular Mylar membranes with the standard thickness of 10 mil (1 mil = 0.001 inch) arranged together to the total thickness of 40, 80 and 150 mil, to generate shock waves with three discrete peak overpressures (approximately 70, 140 and 210 kPa) in the test section (T5 sensor, Fig 1). All tests were performed at room temperature.

### Pressure measurement, headform preparation, and instrumentation

**Fig 2:**
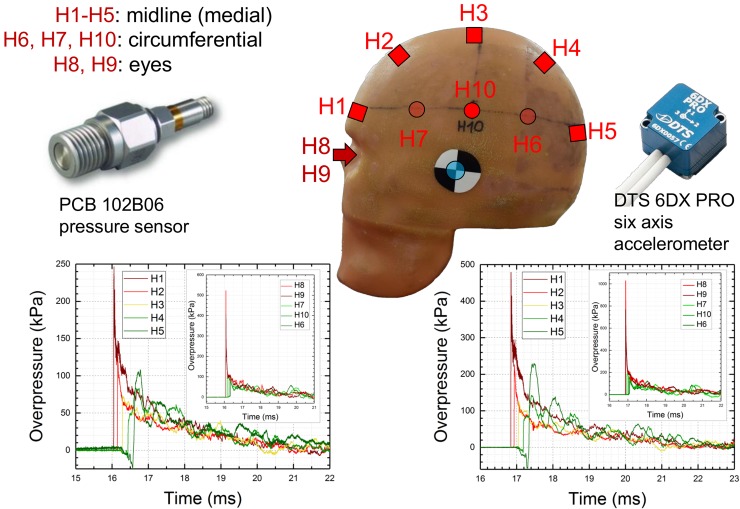
The schematic representation of the headform pressure sensor distribution. The PCB 102B06 sensors were used exclusively: the five medial sensors H1-H5 are mounted at 45 degrees intervals along the midline, while additional sensors are mounted on the circumference: H6, H7 on the right side, H8 and H9 in the eye cavities and H10 on the right side. The DTS 6DX PRO six-axis accelerometer is mounted inside of the headform in the center of gravity. Representative pressure profiles recorded by surface pressure sensors at nominal BOPs of 70 kPa (left, bottom) and 140 kPa (right, bottom) are presented.

The overpressure of incident (side-on, static) blast waves was measured using a series of seven high frequency pressure sensors model 134A24 (PCB Piezotronics, Inc., Depew, NY), which were distributed along the shock tube (Fig 1). Incident static pressure on the outside was measured using pencil probe ICP® 137B24B (PCB Piezotronics Inc., Depew, NY). The FOCUS headform [31], was instrumented with 10 PCB Piezotronics (Depew, NY) model 102B06 pressure sensors: five medial sensors, denoted as H1 to H5, are located along midline anterior-posterior in 45° intervals, and five circumferential sensors mounted in the following order: two on the right parietal side (H6 and H7, in 60° degrees intervals), two in both eye sockets (H8 and H9) and one on the left parietal side of the headform (H10, 90° interval, Fig 2). These sensors were mounted flush to the surface using tapped holes. The six-axis accelerometer DTS 6DX PRO (Diversified Technical Systems, Seal Beach, CA) was mounted in the center of gravity. A LabView program running on a data acquisition system based on National Instruments PXI-6133 S Series multifunction DAQ modules and PXIe-1082 PCI Express chassis was used to capture the signal. All data were recorded at 1.0 MHz sampling frequency and the typical acquisition time was 50 milliseconds. The signal of pressure sensors was fed through 8-channel signal conditioners PCB 483C05 (PCB Piezotronics Inc., Depew, NY) and did not require additional filtration. The signal from accelerometers was filtered with 9.5 kHz low pass filter.

The headform was mounted on the Hybrid III neck (Humanetics, Plymouth, MI)[32], in a rigid configuration to eliminate the motion of the headform during shock wave impact. The FOCUS headform-Hybrid III neck the assembly was attached to the adapter plate and bolted to the bottom of the shock tube in the test section in three different locations (Fig 1).

### Experimental design

The study was designed as two-factor experimental design, 3 x 3. Two experimental variables investigated in this study are 1) the headform location (three levels: inside, end and outside, Fig 1) and 2) shock wave intensity (three levels: 70, 140 and 210 kPa). The incident blast overpressure was controlled by adjusting the thickness of Mylar membranes sandwiched between the breech and expansion section. The thickness was adjusted by stacking individual membranes with thickness of 10 mil (0.01 inches, or 0.254 mm). All tests were repeated four times (n = 4) per nominal shock wave intensity and headform location.

### Data reduction and statistical analysis

All waveforms were imported, processed and quantified in Origin 2017 software (OriginLab Corp., Northampton, MA). Data from experiments performed at different experimental conditions (shock wave intensity and headform location) were pooled together in 3 subsets according to blast intensity (membrane thickness).

The normalization and data reduction procedure were performed as follows. The tabulated values of the four characteristics were divided by their equivalents of the incident shock wave e.g., BOP_H1_ /BOP_T5_ will yield normalized BOP value for the inside of the shock tube (T5 location). Similarly, the normalization was performed for the remaining three parameters: rise time, duration and impulse (Eq 1):

$$x_{non}=\frac{x_{p}}{x_{i}}$$ (1)

where: *x*_*p*_—peak overpressure, rise time, duration or impulse of the resulting pressure waveform on the headform, *x*_*i*_—peak overpressure, rise time, duration or impulse of the incident shock wave.

Multiple comparison, two-tailed t-test was performed with Bonferroni correction and p < 0.003 was considered statistically significant. All data are presented as mean and standard deviation.

## Results

### Incident shock wave characterization

In the initial phase of this project we had adjusted the breech volume necessary to generate three shockwave intensities approximately 70, 140 and 210 kPa peak overpressure in the test section (T5 sensor, Fig 1). The duration was adjusted to be in the range of 5–7 milliseconds (Fig 1). We tracked the evolution of the shock wave profile using a set of 8 sensors: seven of these sensors were mounted on the shock tube wall (A2 to D7, Fig 1) and a single additional one was a pencil probe and mounted on a platform just 0.14 m (approx. 5.5 inches) outside of the shock tube mouth (denoted as PP in Fig 1).

**Fig 3:**
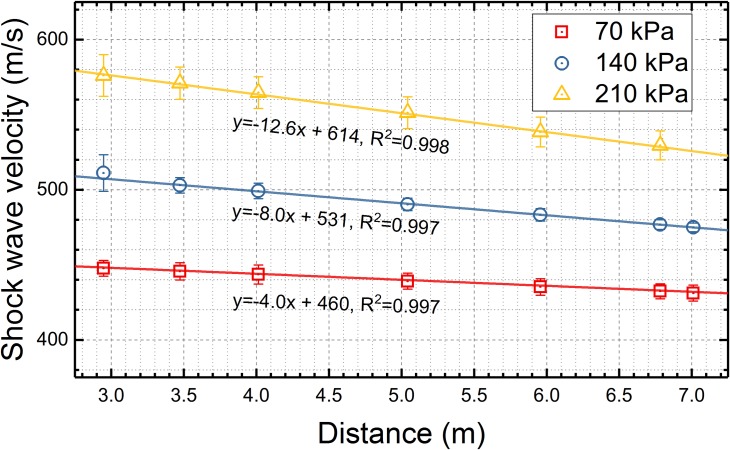
The incident shock wave velocities. The shock wave velocities were calculated at different sensor locations at three discrete BOPs, 70, 140 and 210 kPa, used in this study. Shock wave velocities were averaged for twelve individual measurements, and average values were fitted using the linear function. For a diagram of sensor distribution and dimensions refer to Fig 1.

The velocity of the incident shock wave was measured using arrival times with the A2 sensor as a reference. In these experiments, headform was not present in the test section. For all three nominal shock wave intensities, the shock wave velocities along the shock tube decrease proportionally with the distance from the membrane mounting port and the velocity decay follows linear function (Fig 3). The quality of the linear function fit is excellent in all three cases based on the R^2^ values, which are above 0.99. Velocity at the outside location for 210 kPa nominal shock wave intensity was not measured.

**Fig 4:**
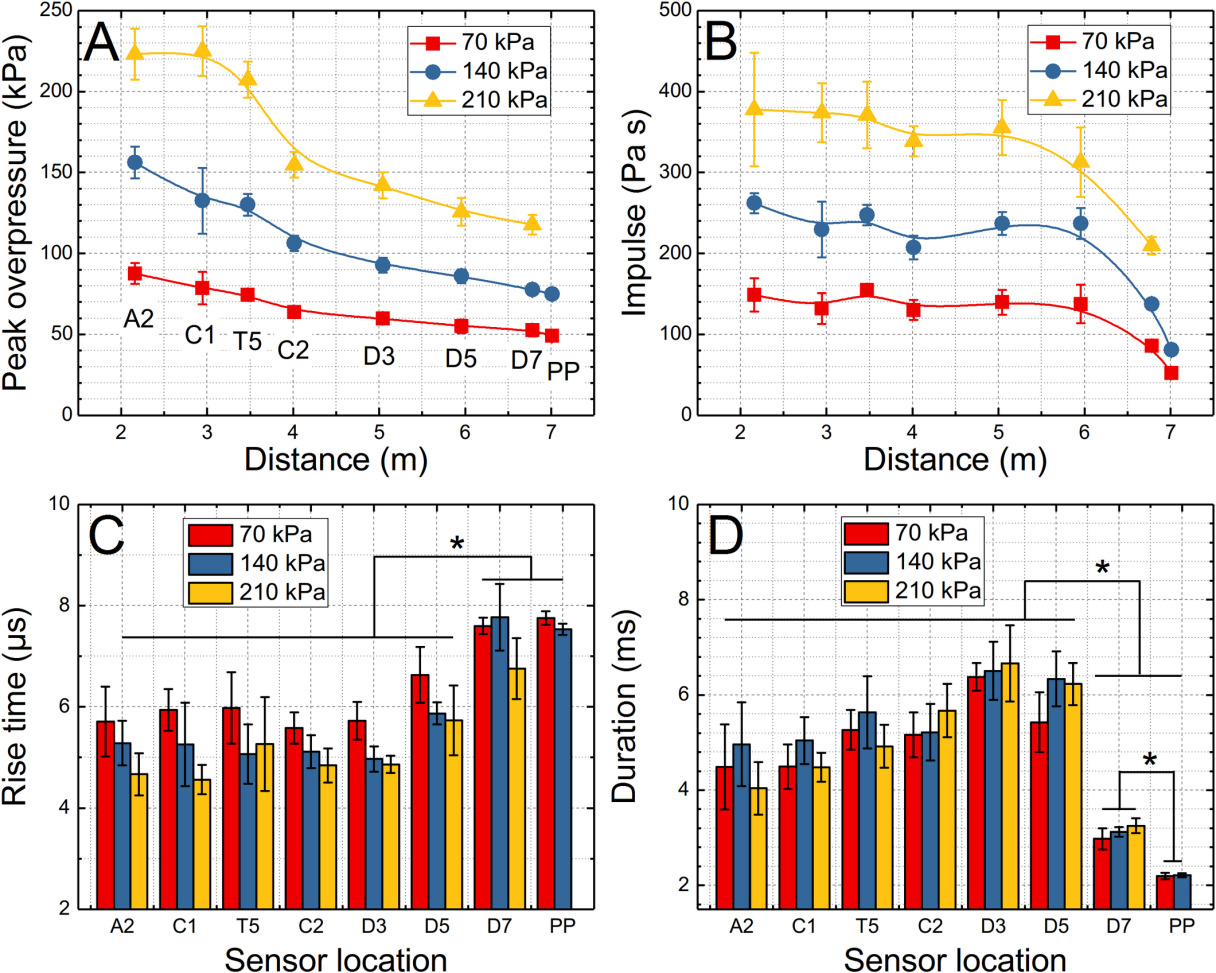
Quantification of four characteristics of the incident shock wave. The peak overpressure (A), impulse (B), rise time (C) and duration (D) for three nominal BOPs used in the experimental design are presented. The evolution of the peak overpressure and impulse values is expressed as a function of sensor distance from the membrane mounting port. The bar plots were used to illustrate the evolution of the rise time and duration as a function of sensor location and BOP. Asterisk indicates the statistical significance exists between respective groups shown in both plots.

We quantified the four characteristic parameters of the shock wave: peak overpressure, rise time, impulse and duration (Fig 4) to gain insight into their spatiotemporal evolution and to establish a baseline for comparison with pressures reported by sensors mounted on the headform (Fig 2). The peak overpressure decreases in a non-linear fashion in all three shockwave intensities used (Fig 4A). The rise time values are in the 4 to 6 microseconds range for sensors located further away from the exit (from A2 to D3, 2 meters upstream from the exit) independently on the shock wave intensity, but there is a noticeable trend of decreasing rise time with shock wave intensity at any specific location (Fig 4C). At the D5 location (1 meter upstream from the exit) the rise time is significantly increased and this trend continues in D7 and PP locations with where the rise time values are exceeding 7 microseconds (6.8 for 210 kPa BOP). The impulse values remain relatively stable for up to 6 meters from the membrane location (which corresponds to D5 sensor location). At the end (D7) and outside (PP) of the shock tube, the impulse values are severely reduced, which is associated with a reduction of the shock wave duration (Fig 4D). The duration values are initially relatively short, i.e., in the 4–5 ms range for the A2 sensor and gradually increase to reach a maximum value at the D3 location (6.2–6.3 ms range). Then somewhat reduced values can be noticed at the D5 location, which is followed by a severe decrease to 2.8–3 ms (D7) and 2.1 ms on the outside.

### Surface pressure on the headform

**Fig 5:**
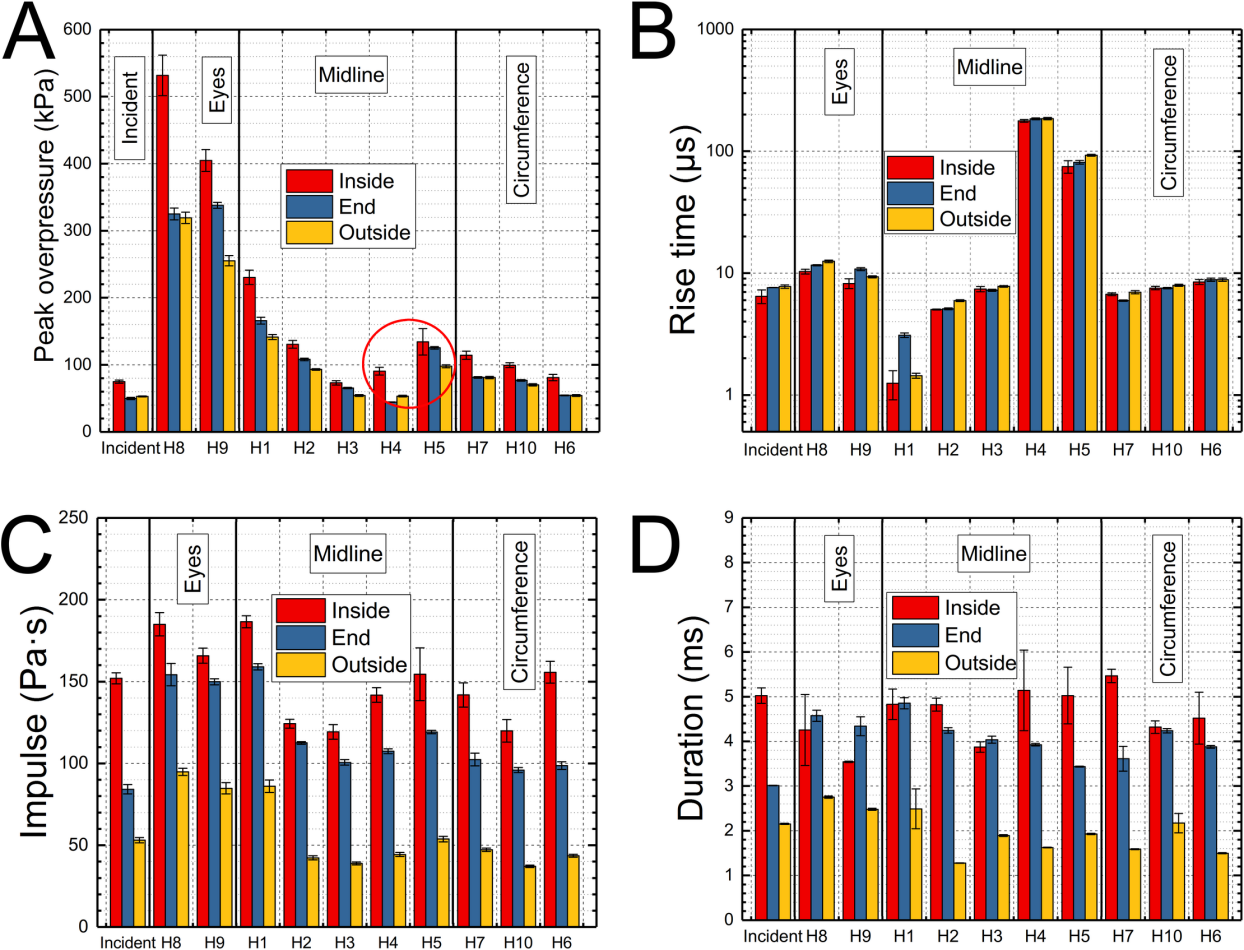
The quantification of four pressure wave characteristics for the headform exposed to at a nominal incident shock wave intensity of 70 kPa. The quantification of peak overpressure (A), rise time (B), impulse (C) and duration (D) recorded for a nominal BOP of 70 kPa. The average values (n = 4) in bar plots were grouped into the four following categories concerning sensor location: incident, eyes (H8 and H9), midline (H1 to H5) and circumference (H6, H7, and H10). For the quantification of the data recorded at 140 and 210 kPa BOPs refer to Supporting information, S2–S5 Figs in the supporting information.

Representative pressure profiles recorded by the surface sensors mounted on the headform are presented in Fig 2. The sensors located on the front face of the headform (H1, H2) and in the eye sockets (H8, H9) report the highest pressures, irrespectively of the nominal shock wave intensity. Interestingly, the H5 sensor located on the back of the headform records significantly elevated pressures compared to the nominal shock wave intensity. In Fig 5 the results of the surface pressure measurements at various locations on the headform are summarized. We quantified four characteristic parameters of the shock wave: 1) peak overpressure (Fig 5A), 2) rise time (Fig 5B), 3) impulse (Fig 5C), and 4) the duration (Fig 5D). The results in the bar plots are categorized according to the sensor location: 1) eyes (H8 and H9), 2) midline (H1 to H5) and 3) circumference (H6, H7, and H10). Incident shock wave characteristics for all three test locations (inside, end and outside) are also presented. The peak overpressure values vary in the 260 to 540 kPa for eye socket mounted sensors (H8, H9). In general, the headform peak overpressure scales with the peak overpressure of the incident shock wave. For a specific sensor mounted on the headform, the peak overpressure decreases in the order of headform location: inside, end and outside. The rise time for incident shock waves is in the range of 6–8 μs independently on the test location (Fig 5B). The rise time values increase gradually for the midline group: the rise time of the H1 sensor is in the 1–3 μs range, followed by H2 (5–6 μs), H3 (7–8 μs), reaching maximum at H4 (approx. 110 μs) and decreasing somewhat at H5 location (70–90 μs). The eye sensors have the rise times in the range between 8 to 10 μs, while sensors on the circumference report rise times of 6–9 microseconds. The impulse values are the highest for the entire set of pressure sensor locations when the headform was placed in the inside of the shock tube (120–190 Pa·s). In the end and outside locations the impulse values are in the range of 80–160 Pa·s and 40–90 Pa·s, respectively. A similar trend is observed for the duration of the shock wave: inside (3.6–5.4 ms), end (3.0–4.8 ms) and outside (1.3–2.8 ms). Similar trends are true also at higher nominal shock wave intensities (140 kPa and 210 kPa, S2 and S3 Figs, respectively).

### Normalized shock wave characteristics

**Fig 6:**
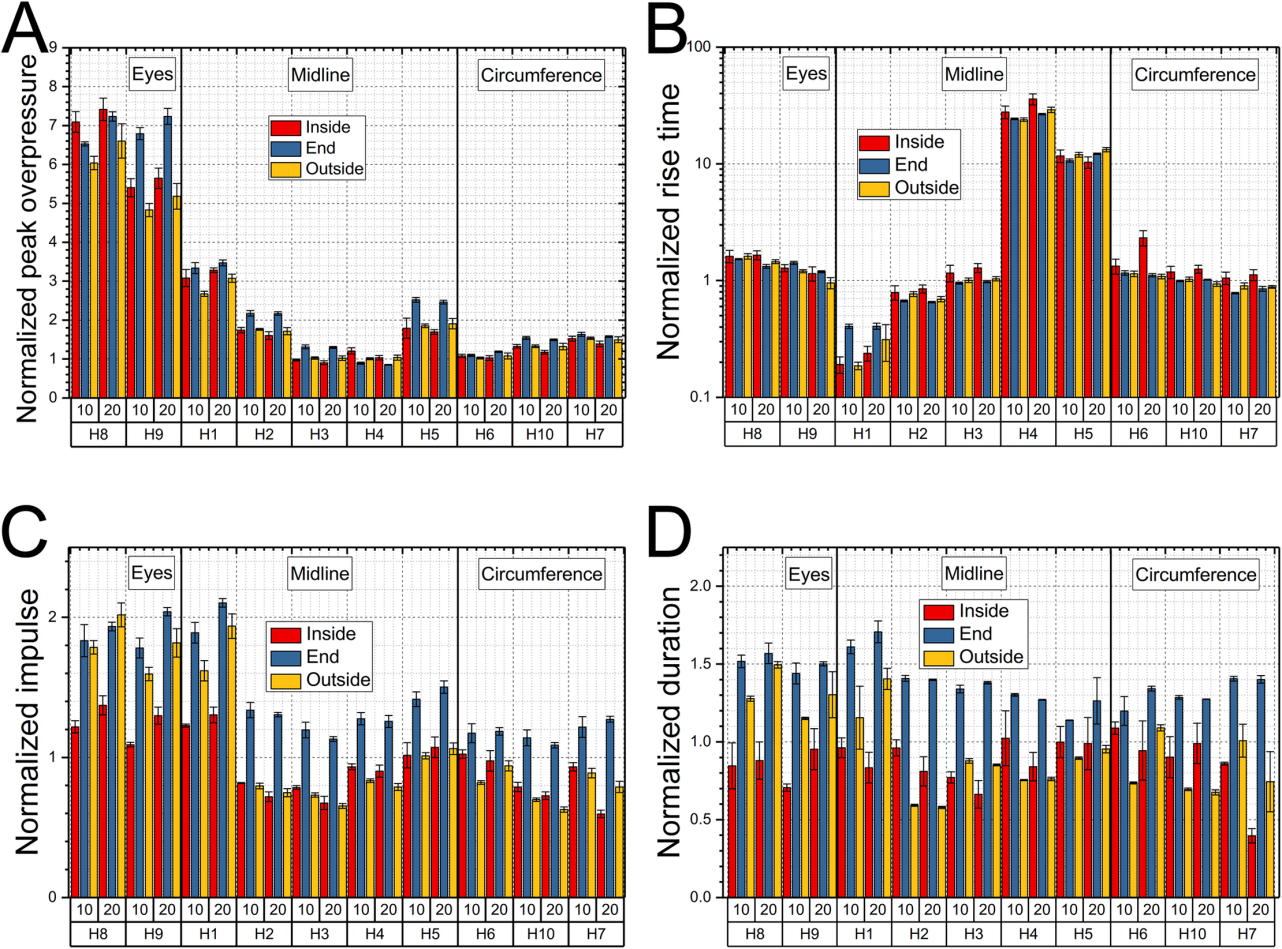
The normalization of the four characteristics of the pressure waveform recorded by headform surface sensors. The bar plots of the normalized peak overpressure (A), rise time (B), impulse (C) and duration (D) for surface pressure profiles recorded at various locations on the headform as a function of shock wave intensity (70 and 140 kPa) and headform location (inside, end and outside). The values for 210 kPa BOP are not included for clarity. The values in bar plots were grouped into the four following categories with respect to sensor location: incident, eyes (H8 and H9), midline (H1 to H5) and circumference (H6, H7 and H10).

The normalized characteristic parameters for two nominal shockwave intensities and three test locations in the shock tube are presented in Fig 6. The normalization procedure for all four characteristic parameters of the shock wave was described in experimental section.

It is evident from Fig 6 that this transformation allows comparison of results obtained at different locations and wide-range of shock wave intensities. The peak overpressure and rise time (Fig 6A and 6B) show a high level of uniformity, with the exception of eye socket sensors (H8 and H9), which have elevated values in the range of 5.0 to 7.0. The normalized peak overpressure for the H1 sensor is around 3.0, and in the 1.0 to 1.6 range for the remaining sensors, with the exception of H2 and H5 where values from 1.8 to 2.4 are apparent.

**Fig 7:**
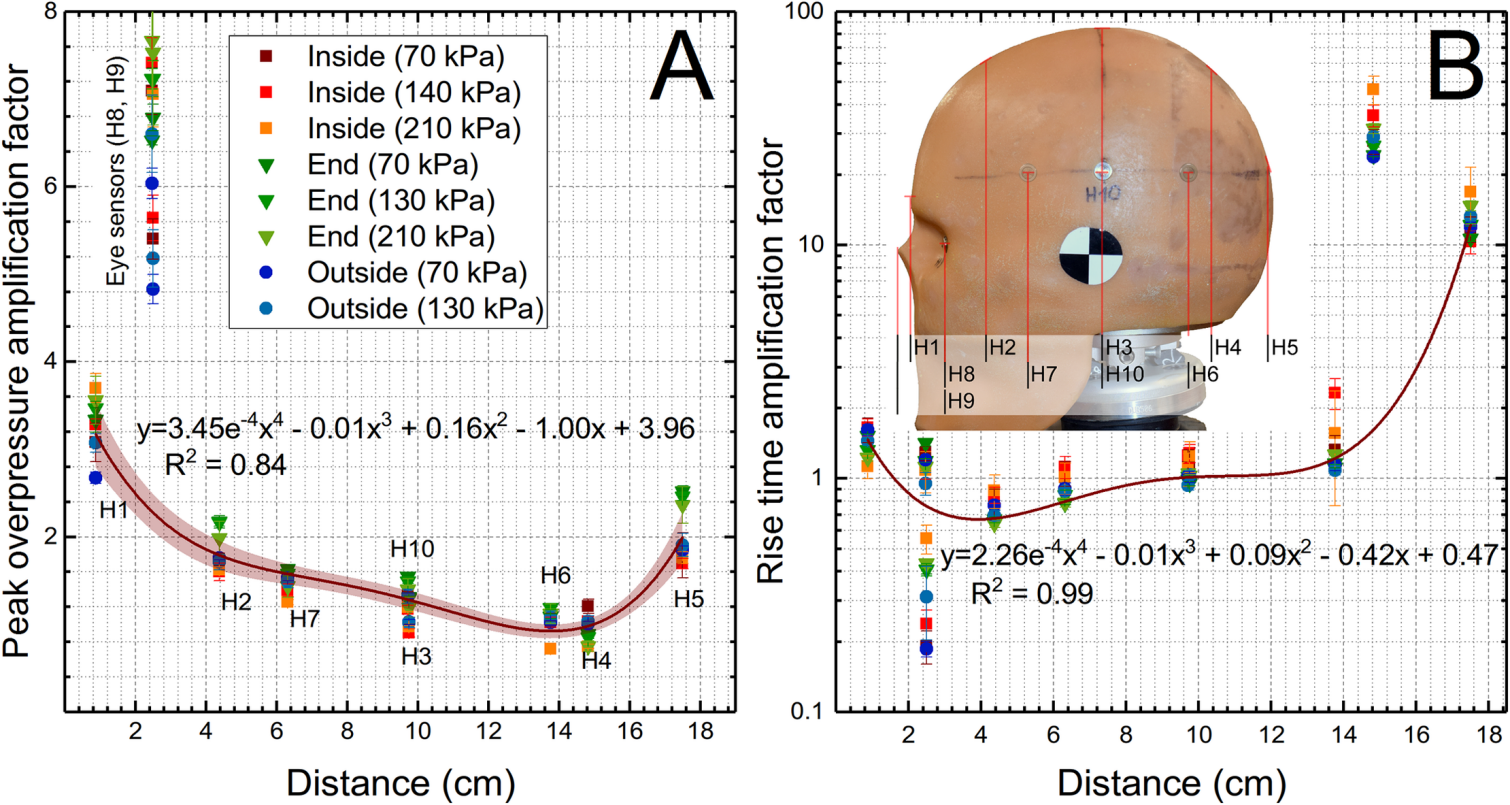
The amplification factors for the peak overpressure and rise time at three nominal shock wave intensities. The plots demonstrating peak overpressure (A) and rise time amplification factors as a function of sensor location along the abscissa of the direction of the shock wave propagation. Normalized values for pressure sensors calculated for measurements performed at 70, 140 and 210 kPa BOPs are presented. Inset: the schematic illustrating the origin and sensor distances used in fits where fourth-degree polynomial functions were used. The H8 and H9 data were excluded from fitting procedure in both cases, while for the rise time the data for H4 sensor were also eliminated.

The most extensive distribution of values is noticeable for the rise time, which spans from 0.2 (H1) to 35 (H4), but for the majority of sensor locations, these numbers are around 1.0 (Fig 6B). The normalized impulse values for front face sensors (H1, H8, and H9) have values in the range of 1.1–1.4 (Inside), 1.8–2.1 (End) and 1.6–2.0 (Outside). The remaining sensors have the values in the range 0.6 to 1.0 (Inside and End) and 1.1 to 1.5 (End). The normalized duration values for the inside location are within 0.7–1.0 range with the exception of H7 sensor (0.4). For the end location the normalized duration values exceed 1.0, and are in the range from 1.2 to 1.7. Normalized duration values for the outside depends highly on the sensor position on the headform and have the largest variation, i.e., from 0.6 to 1.5. The normalized parameters are called amplification-attenuation factors in Fig 7.

The relationships between the peak overpressure and the rise time as a function of respective surface sensors of the distance along the X-axis on the headform (the nose tip is a reference) is presented in Fig 7. We used fourth order polynomial fit to model these data points, and eye sensor data were excluded from the fit with good results (R^2^ = 0.84). Similarly, the fourth order polynomial fit was used for the rise time and in this case data from both eyes and H4 sensors were excluded from the fit to obtain high fit fidelity (R^2^ = 0.99). However, the polynomial fit of the other two parameters, impulse, and duration, give unsatisfactory results (R^2^ = 0.42 and 0.09, for impulse and duration, respectively see S4 and S5 Figs).

## Acceleration

**Fig 8:**
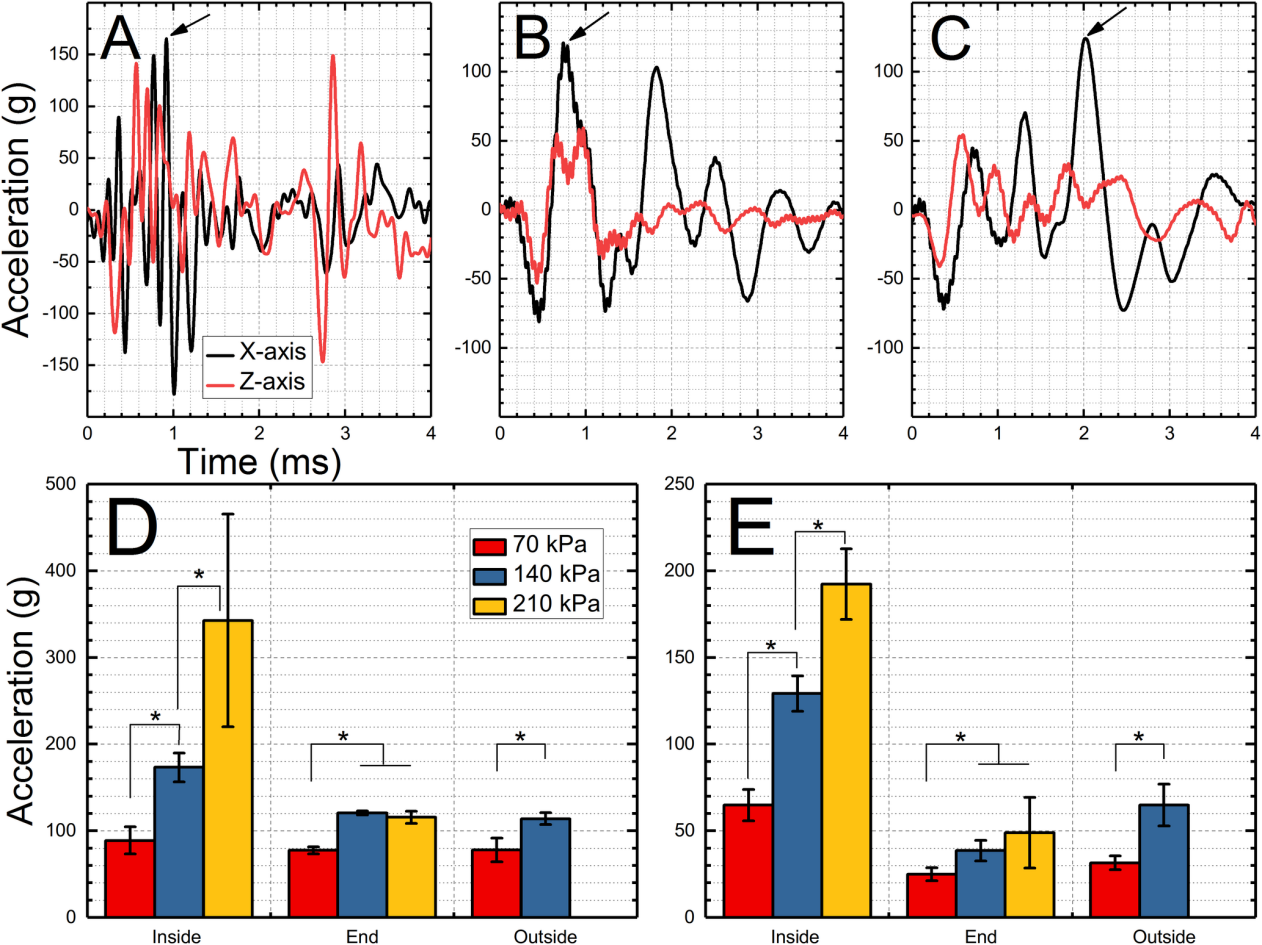
Comparison of the linear acceleration of the headform tested in three different locations. Linear acceleration (X and Z axis) signals for the headform exposed to a 140 kPa nominal intensity shock wave at different locations: A) inside, B) end, and C) outside of the shock tube. The arrival time of the H1 sensor was used as a reference. Quantification of the peak acceleration for the acceleration along the X-axis (D) and Z-axis (E) for three nominal shock wave intensities and three test locations. Asterisk indicates groups with statistical significance threshold p < 0.05.

The representative linear acceleration profiles are presented in Fig 8A–8C (only X and Z axis profiles are presented for brevity). The quantification of the peak acceleration as a function of shock wave intensity and test location is presented in Fig 8D and 8E. The general trend for peak acceleration measured along both axes is that it increases with nominal shock wave intensity for the inside location (p <0.05). The same is true for the end and outside locations independently on the axis of acceleration, where the nominal intensity of 70 kPa results in the lowest acceleration and these results are statistically significant (p < 0.05).

The point of peak acceleration is marked with an arrow in Fig 8A–8C. For the inside and end location, the elapsed time to peak acceleration (ETPA) is nearly the same, i.e., in the 0.7–0.8 range, while for the outside the maximum acceleration is reached after 1.8–2.0 milliseconds (acceleration measured along X-axis, S6A Fig). For the Z-axis acceleration the ETPA is gradually increasing in the order depending on the test location: inside < end < outside (S6B Fig). The ETPA doesn’t seem dependent on the nominal intensity of the shock wave.

**Fig 9:**
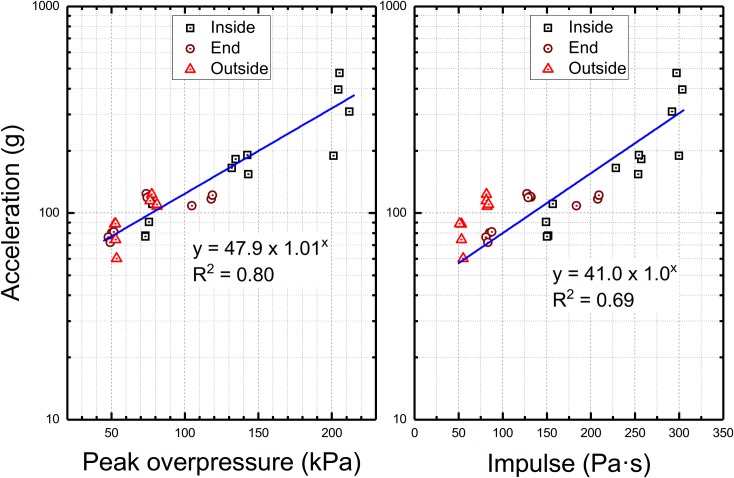
The effect of peak overpressure and impulse on the peak acceleration. The semi-log plots of the peak acceleration as a function of the peak overpressure (left) and impulse (right) measured at the respective test locations. The experimental data points for the Z-axis were fitted using exponential growth function y = a·b^x^.

We evaluated whether the maximum acceleration is a function of shock wave intensity, i.e., peak overpressure and impulse, and the results are presented in Fig 9. It would appear the experimental data follow exponential growth function expressed by the following equation:

$$y=ab^{x}$$ (2)

The values of the goodness of fit parameter, R^2^, indicate this function fits the data with higher fidelity where peak overpressure is used as the abscissa, as compared to impulse (R^2^ = 0.80 and 0.69, respectively, Fig 9). For the acceleration measured along the Z-axis, the values of these parameters increase to 0.87 and 0.91 (S7 Fig) for peak overpressure and impulse.

## Discussion

The motivation of this work is to standardize test parameters for evaluating personal protective equipment (PPE) performance in blast loading conditions. Considering there is no shock tube testing standards a number of approaches are used for this purpose. In the early days, the experimental were frequently performed on the outside or at the end of the shock tube [6, 7, 19, 33]. This approach while not without some merit is a convenience-driven rather than rational design to replicate true field conditions [34, 35], with a possibility of the significant contribution of dynamic loading [36]. The testing of the specimen with the dimensions of the human head requires a broad cross-section shock tube to be employed, which otherwise would result in the pressure artifacts associated with the blockage [36]. However, a literature survey of military PPE evaluation methodologies indicates most of the work in this area relies heavily on numerical simulations evaluating the protective effect of helmets [6, 8, 21, 23, 25, 28, 37] including helmet performance under ballistic impact [10–12]. Numerical simulations are an invaluable tool for to examine mechanistic aspects of blast induced neurotrauma [18], effects of existing and new padding materials [8] and helmet designs [21, 28] on the pressure attenuation and transmission pathways [23, 25], among others. However, their primary limitation from the perspective of quality control of PPE is lack of necessary fidelity when confronted with basic materials imperfections and variability of their properties [38]. It’s been also demonstrated that simple tasks like fitting the helmet model with pads to the anthropometric head phantom might pose a substantial challenge during model preparation step [7]. For day-to-day operations in the research facility, it is just impractical and time-consuming, while simultaneously lacking in information about material properties (especially true for proprietary foam designs used in padding materials) and defects leading to the variability of the experimental results. With that in mind, we employed in the initial step of this project a comprehensive characterization of the headform in the shock tube.

The evaluation of the relationship between different test locations was performed using the headform mounted in three different places: inside, at the end and on the outside of the shock tube (Figs 1 and 2). These three test locations are typically used in the laboratory shock wave testing [7, 22, 38–41], but flow fields generated in these places vary significantly [34, 42]. Growing body of evidence suggests outside location is not representative of the field conditions associated with high explosive blast waves [36].

We first evaluated the evolution of the shock wave traveling in the test section and quantified four characteristic parameters: peak overpressure, rise time, duration and impulse (Fig 4). The peak overpressure for all 3 nominal shock wave intensities decreases monotonically, accompanied by declining shock wave velocity (Figs 3A and 4). It is typically observed in the shock tubes with similar length when a sufficient number of pressure sensors is employed to track the progression of the shock wave [34, 42]. Similarly, the duration of the incident shock wave increases, which is accompanied by a concomitant increase in the rise time However, the shock wave duration is reduced severely by the rarefaction at the exit of the shock tube, while the rise time of the shock front continues to increase even for the outside location. Considering the impulse values are correlated primarily with the duration it is evident that the sharp decrease in impulse values is observed at the end and on the outside.

The experiments with the headform were executed in the next step, which was followed by quantification of the four characteristics of each pressure waveform reported by ten surface mount sensors (Fig 2). The highest peak overpressures are observed for the eye socket sensors and forehead mounted sensor (Figs 2, 5 and 6), which was also reported by others using numerical models [7, 37, 41]. The pressure profiles of all surface sensors closely resembles the incident shock wave profile: there is obvious peak pressure increase for the front face sensors (H1, H8, and H9), but there is also a short underpressure occurring before the shock front for two midline sensors mounted on the back of the headform (H4 and H5) indicating the flow separation region (not reported in earlier studies using this headform [6, 20]). The rise times display relative invariance concerning the headform location, and the larges difference is for the H1 sensor: 1.2 ms (inside), 3 ms (end) and 1.5 ms (outside). However, it is apparent the duration and impulse values are strongly dependent on the test location (Fig 5C and 5D). The same general observations are valid for tests performed at 140 and 210 kPa (S2 and S3 Figs).

It is thus apparent a further data reduction is necessary to compare data collected at three different intensities and three different headform locations. The most intuitive approach is to take the characteristic parameters of the incident shock wave waveform (input) recorded at a specific area, i.e., T5 for inside, D7 for the end and PP for outside position, and compare them with waveforms recorded by pressure sensors on the headform (output). The resulting dimensionless parameters are a measure of the disturbance caused by the introduction of headform into the flow field of the shock wave traveling in the shock tube. The values other than 1 indicate divergence from the incident waveform characteristics at the specific test location, and in the particular position on the headform, compared to the incident shock wave. These can be attributed to geometric factors, changes in shock wave characteristics and presence of additional high velocity flows. We demonstrated the shock wave properties are gradually evolving while traveling in the shock tube (Fig 3), and it is reasonable to expect the similar distribution of non-dimensional parameters as a function of their physical location on the headform.

These non-dimensional parameters become the only defining parameter of the system and depend only on the characteristics of the incident pressure waveform. The non-dimensional parameters (amplification factors) allow for direct comparison of pressure waveform characteristic parameters generated by a range of incident shock waves differing in intensity. With this concept in mind, we performed further data reduction for peak overpressures, rise time, impulse and durations in all datasets (Fig 6). The amplification factors for peak overpressures have relatively narrow distribution and follow clear trend independently on the test location. The first sensor H1 has the highest values in the range of 2.4 to 3.6, which gradually decrease along the headform reaching minimum values of about 1 for the H4 and H6 sensors on the back of the headform, and reaching a value of 2 for the H5 sensor at the very end of the headform. This increase is purely due to combined effect of the shock wave wrapping around the headform and its two streams converging at the back. It is accompanied by increased duration of the rise time for the H5 sensor by a factor of 10 compared to the incident shock wave, which is markedly higher compared to all other sensors, where the rise time amplification factor never exceeds the value of 2. Amplification factors for sensors mounted in the eye sockets are very high (in the range of 5–8), and they do not follow the same trend as the other sensors. The concave geometry of eye sockets is responsible for the compressed air entrapment and stagnation during shock wave exposure, which results in extreme pressures [7], compared to other locations on the headform. It would appear that both normalized peak overpressures and rise time follow the well-defined trend as a function of the sensor location on the headform (Fig 7). However, the same is not true for the duration and impulse (S4 and S5 Figs), which exhibit robustly test location dependence.

We have also quantified the linear acceleration along the X- and Z-axis (Fig 8). In both instances, the peak acceleration correlates strongly with incident peak overpressure (Fig 9) and follows exponential function (Eq 2). However, the elapsed time to peak acceleration (S6 Fig) is dependent on the test location. The acceleration persists only for short durations of time (~5 ms), and the headform deflection was usually less than 4 degrees, based on the analysis of high-speed video footage. These peak acceleration values are below 5% head injury threshold [43]. Moreover, relatively large acceleration values were observed in spite of the rigid configuration of the Hybrid III neck used in this study, which is caused by the rubber parts, which constitute this neck. We wanted to eliminate the possibility that material properties of the rubber might change during the experiments, which was a definite possibility considering exposure to 210 kPa uncontrollably deflects the headform. The deflection of the headform is fast enough to affect the pressure distribution on the headform, which was obvious during the preliminary phase of our studies (data not shown).

## Conclusions

We performed a mapping of the pressure distribution using 10 pressure sensors on the headform exposed to a shock wave with three intensities (70, 140 and 210 kPa) and in three different test locations: inside, end, outside. We demonstrated that peak overpressure on the headform can be predicted with reasonable accuracy based only on the peak overpressure of the incident shock wave and is independent of the headform location. Similarly, the rise time can be calculated based solely on the rise time of the incident shock wave. The analysis of the duration and impulse amplification factors indicates these two parameters do not follow simple relationships and their values strongly depend on the test location. Notably, the end location divergence compared to other data is noticeable and consistent across all three shock wave intensities used in this study. A simplified headform model used in this study is suitable for mapping accurately the surface pressure field, which is a critical step to establish a baseline values for evaluation of PPE efficiency. The extension of this work, which is currently in progress, will include evaluation of blast mitigation of currently existing designs of combat helmets, and the development of corresponding numerical models.

## Supporting information

S1 Fig Details of the headform instrumentation with PCB 102B06 pressure sensors. (PNG)

S2 Fig Quantification results of the peak overpressure for 10 pressure sensors mounted on the headform and exposed to shock wave with nominal BOP of 140 kPa (approx. 20 psi). Tests were performed at three locations: inside, end and outside of the shock tube.
(PNG)

S3 Fig Quantification results of the peak overpressure, rise time, impulse and duration for 10 pressure sensors mounted on the headform and exposed to shock wave with nominal BOP of 210 kPa (approx. 30 psi). Tests were performed at two locations: inside and the end of the shock tube.
(PNG)

S4 Fig The impulse amplification factor as a function of sensor location along abscissa of the direction of the shock wave propagation (see Fig 7 for details). Normalized values for pressure sensors calculated for measurements performed at 70, 140 and 210 kPa BOPs are presented. All data points are included in the fit.
(PNG)

S5 Fig The duration amplification factor as a function of sensor location along abscissa of the direction of the shock wave propagation (see Fig 7 for details). Normalized values for pressure sensors calculated for measurements performed at 70, 140 and 210 kPa BOPs are presented. All data points are included in the fit.
(PNG)

S6 Fig Elapsed time to peak acceleration along the X-axis (A) and Z-axis (B) as a function of nominal shock wave intensity and test location (inside, end and outside).
(PNG)

S7 Fig The semi-log plots of the peak acceleration as a function of the peak overpressure (left) and impulse (right) measured at the respective test locations. The experimental data points for the Z-axis were fitted using exponential growth function y = a·b^x^.
(PNG)
