# Supplementary figures and images for: Effective testing of personal protective equipment in blast loading conditions in shock tube: Comparison of three different testing locations
Source: PLoS One. 2018 Jun 12;13(6):e0198968. doi: 10.1371/journal.pone.0198968 (PMC5997325; doi:10.1371/journal.pone.0198968)

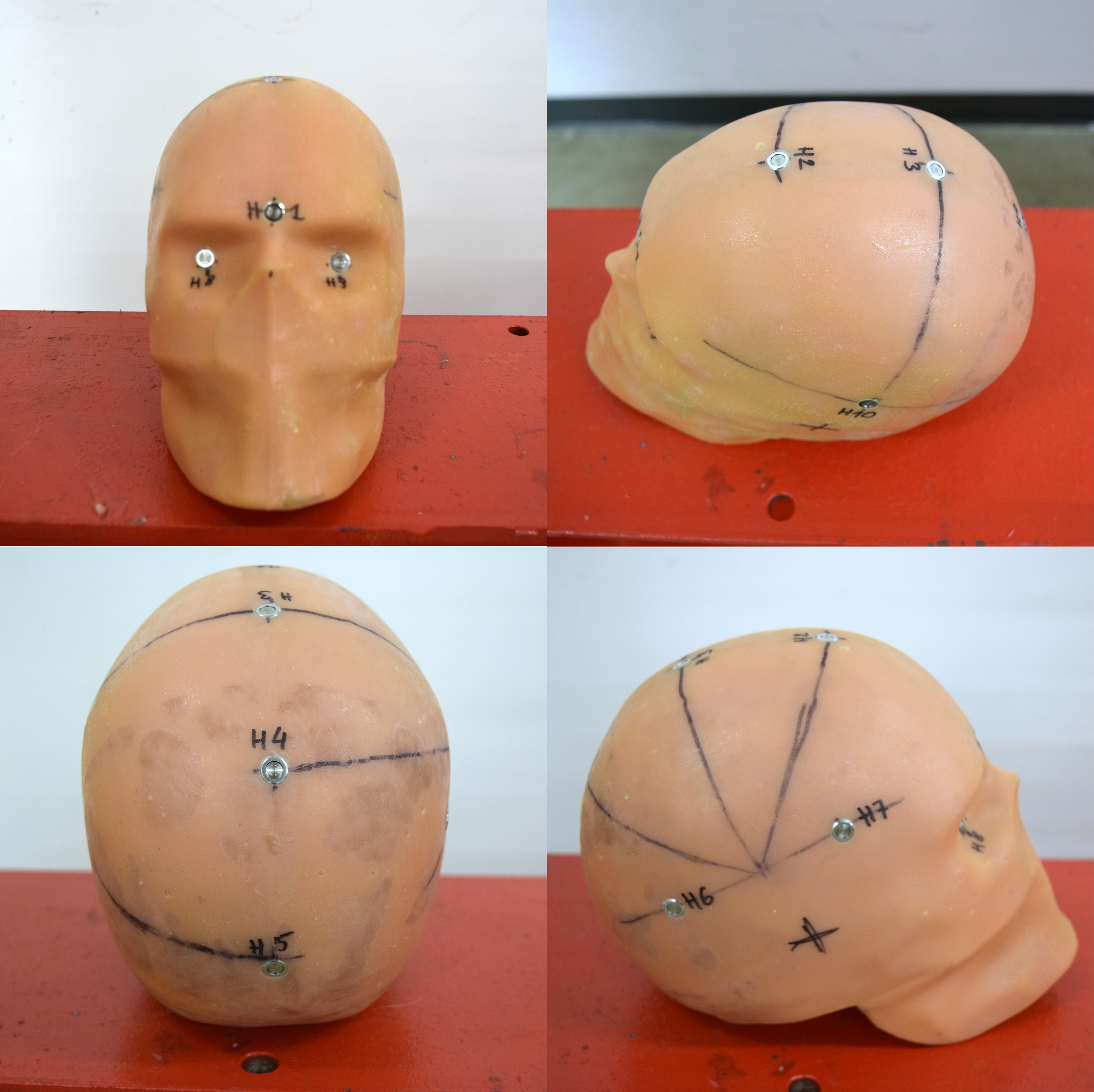

Supplement: S1 Fig — (PNG) [file pone.0198968.s001.png]

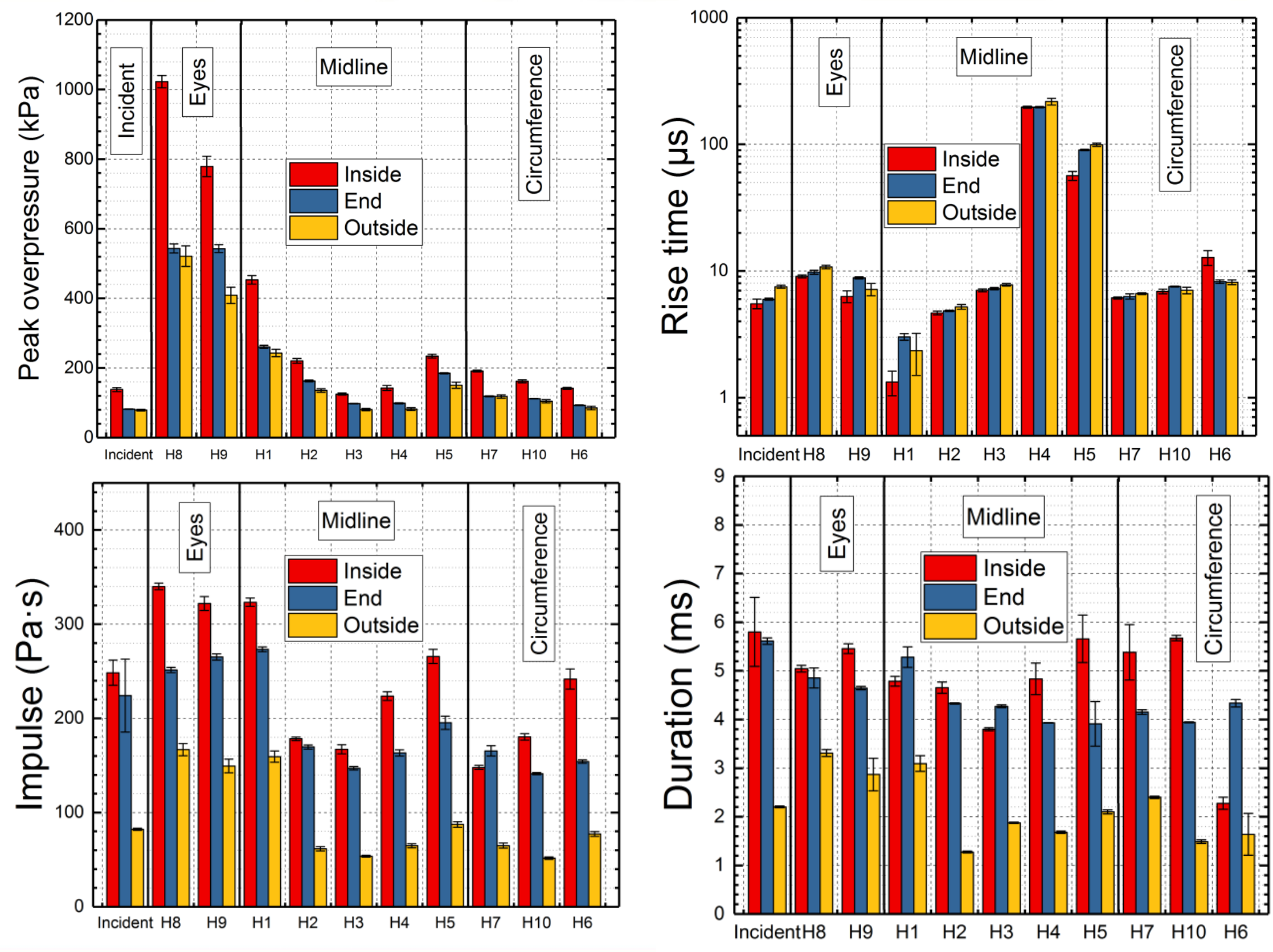

Supplement: S2 Fig — Tests were performed at three locations: inside, end and outside of the shock tube. (PNG) [file pone.0198968.s002.png]

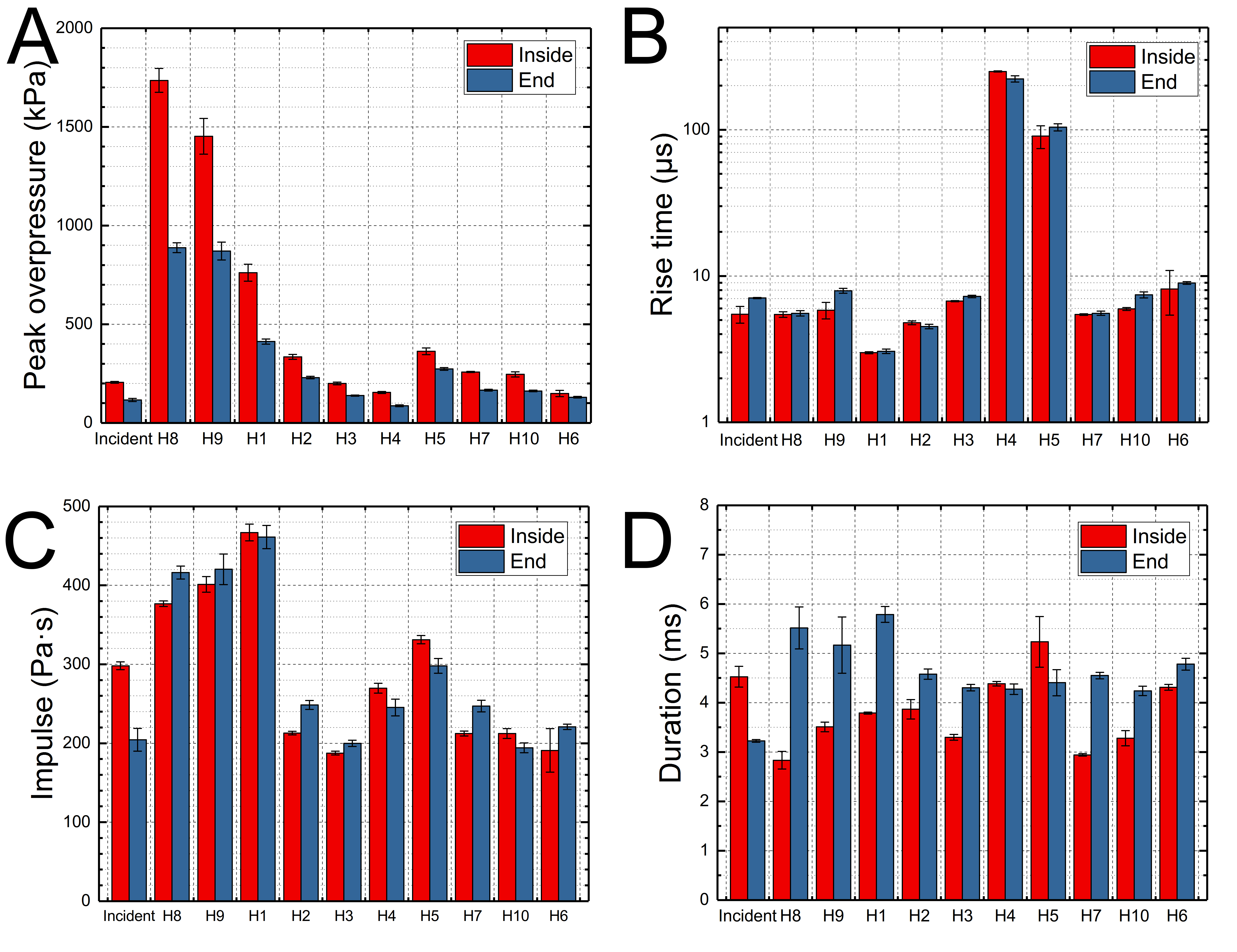

Supplement: S3 Fig — Tests were performed at two locations: inside and the end of the shock tube. (PNG) [file pone.0198968.s003.png]

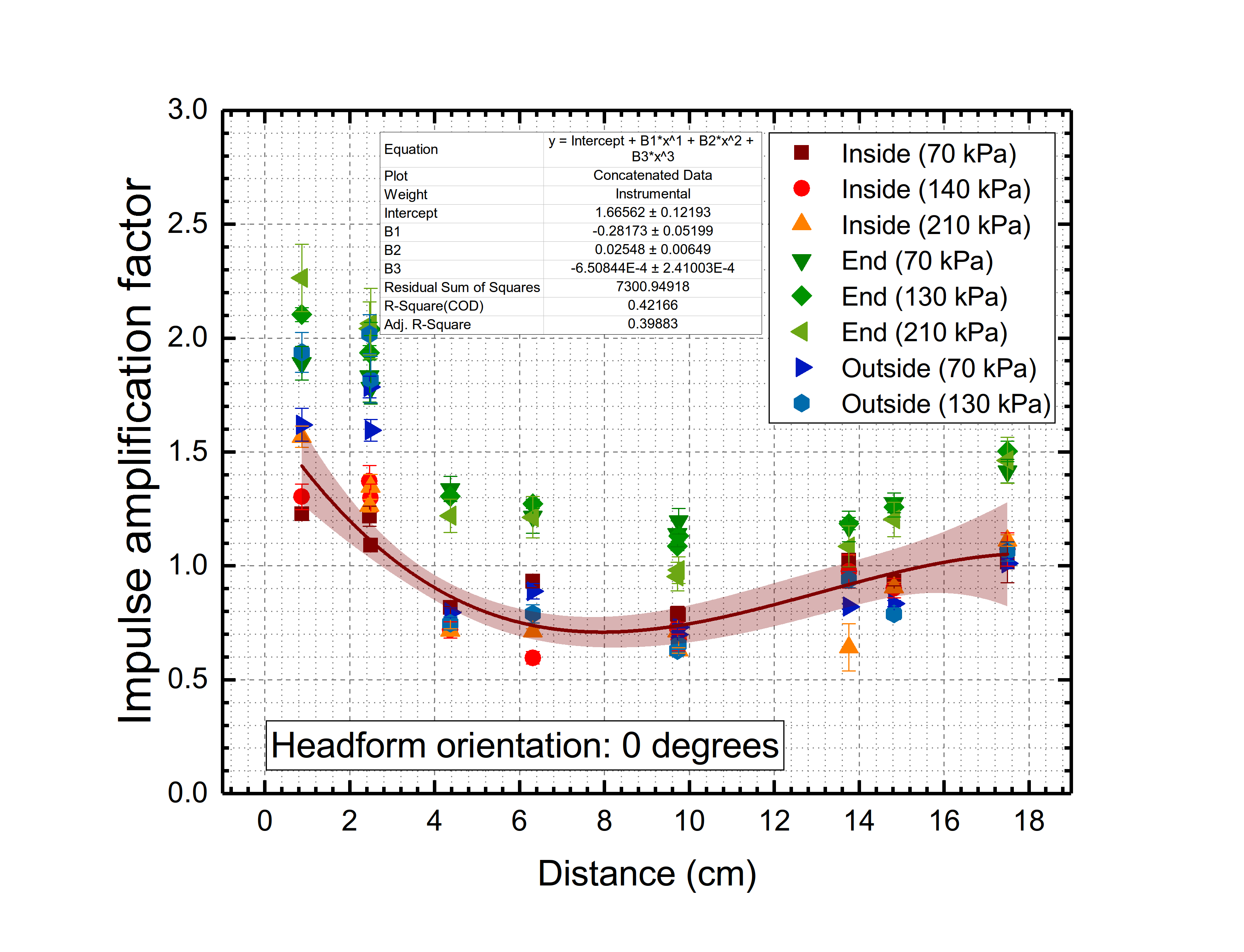

Supplement: S4 Fig — Normalized values for pressure sensors calculated for measurements performed at 70, 140 and 210 kPa BOPs are presented. All data points are included in the fit. (PNG) [file pone.0198968.s004.png]

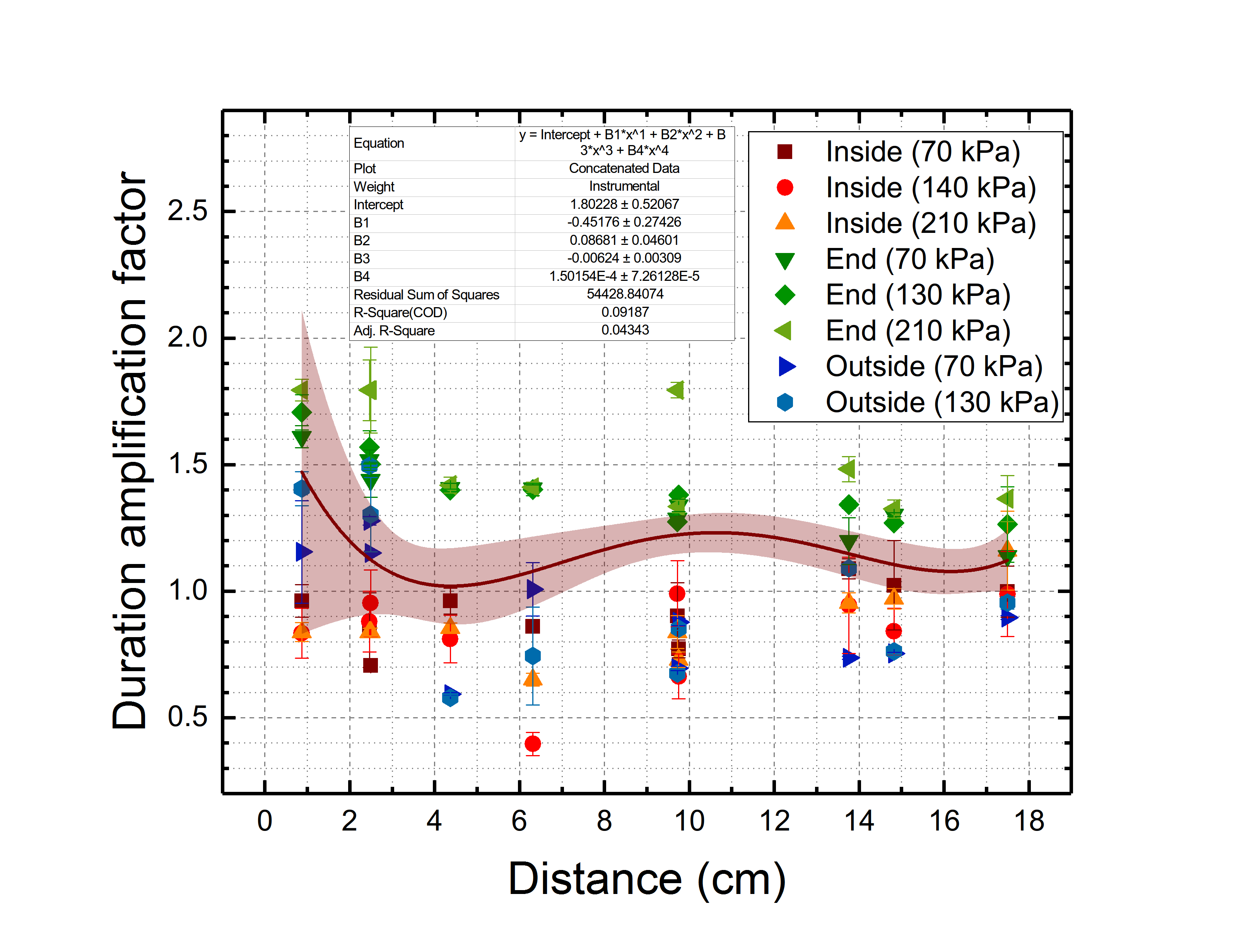

Supplement: S5 Fig — Normalized values for pressure sensors calculated for measurements performed at 70, 140 and 210 kPa BOPs are presented. All data points are included in the fit. (PNG) [file pone.0198968.s005.png]

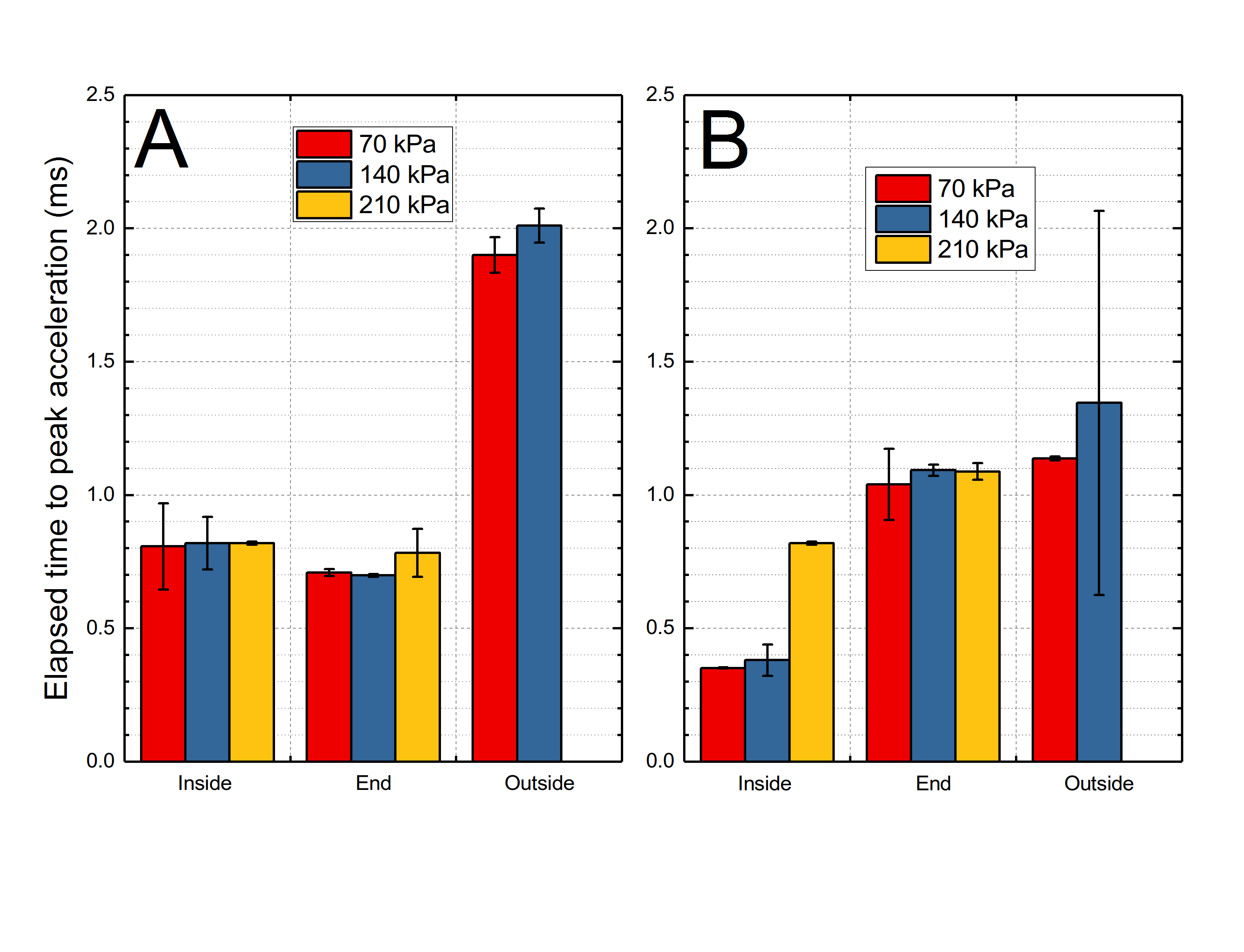

Supplement: S6 Fig — Elapsed time to peak acceleration along the X-axis (A) and Z-axis (B) as a function of nominal shock wave intensity and test location (inside, end and outside). (PNG) [file pone.0198968.s006.png]

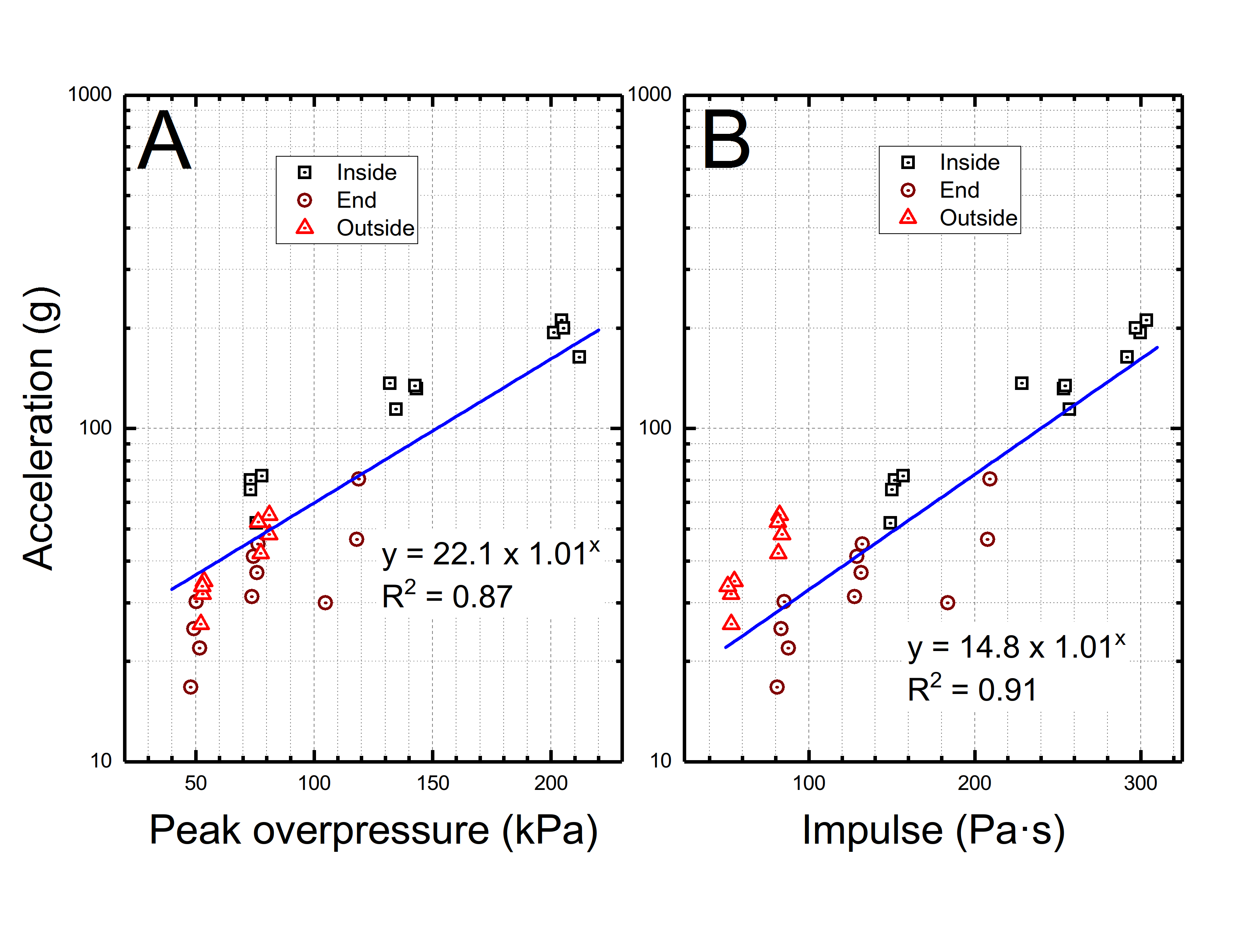

Supplement: S7 Fig — The semi-log plots of the peak acceleration as a function of the peak overpressure (left) and impulse (right) measured at the respective test locations. The experimental data points for the Z-axis were fitted using exponential growth function y = a·bx. (PNG) [file pone.0198968.s007.png]
